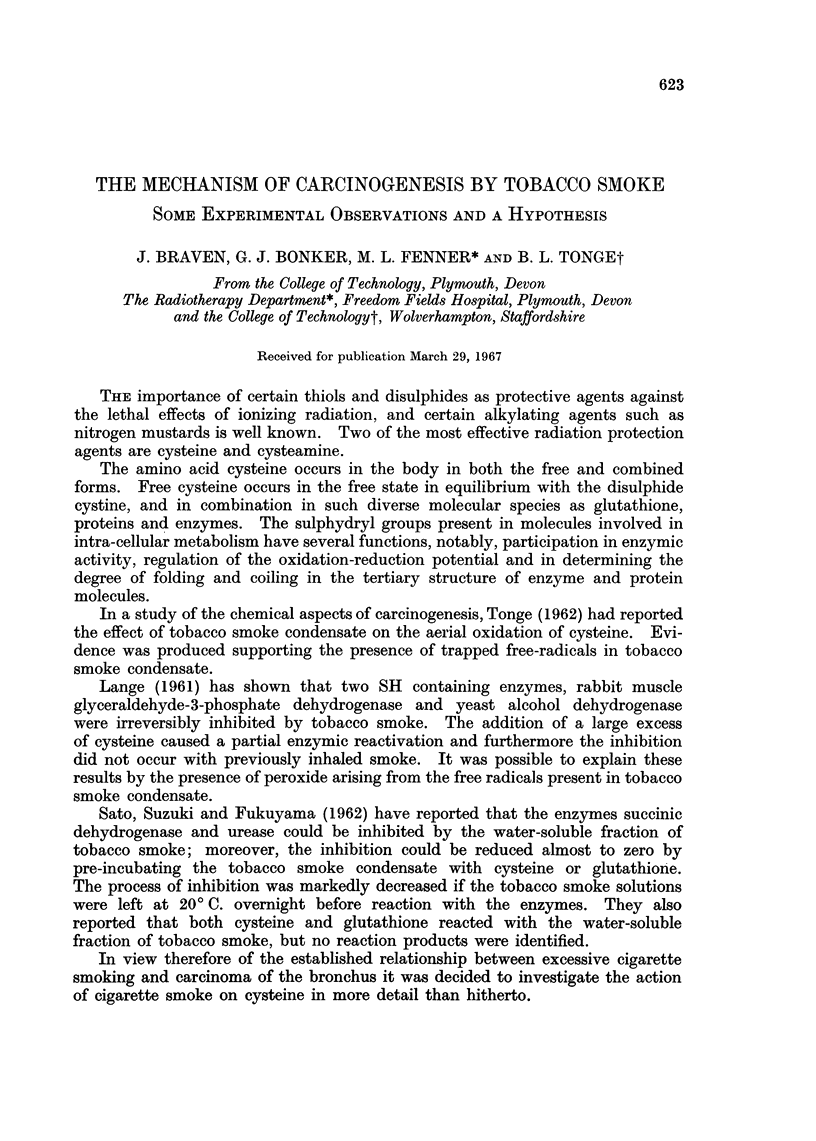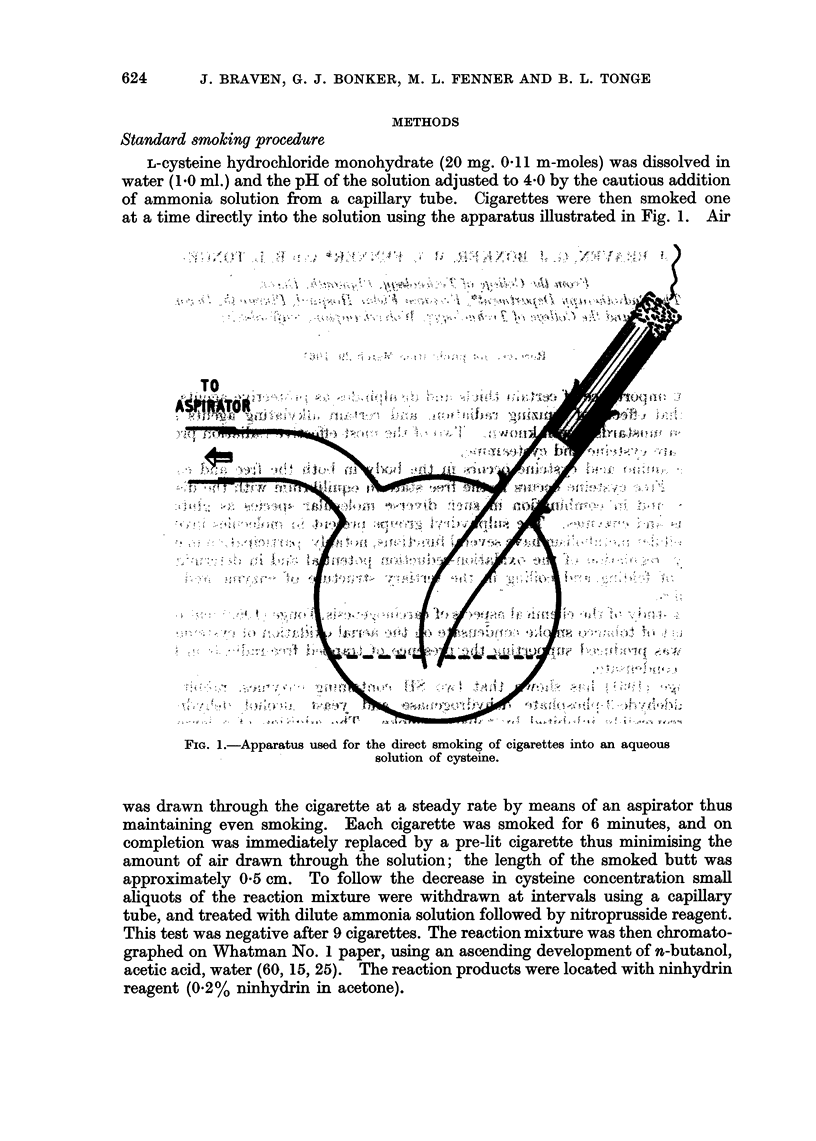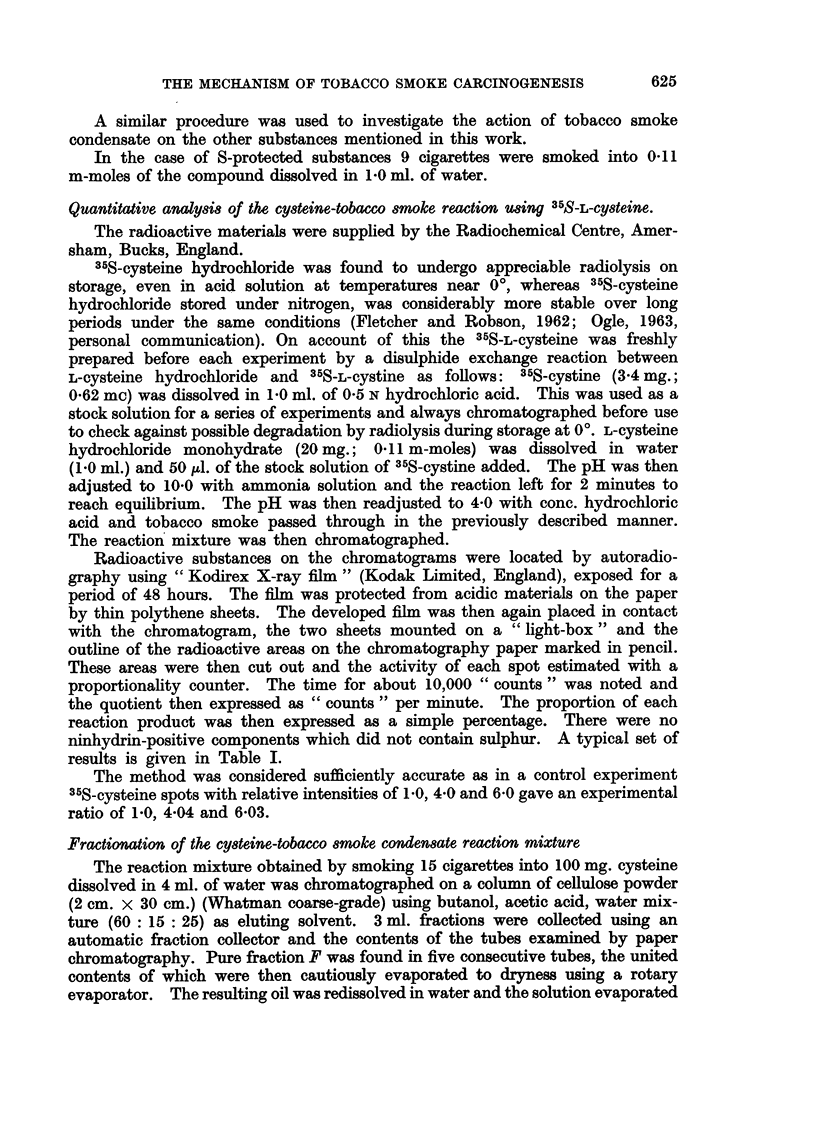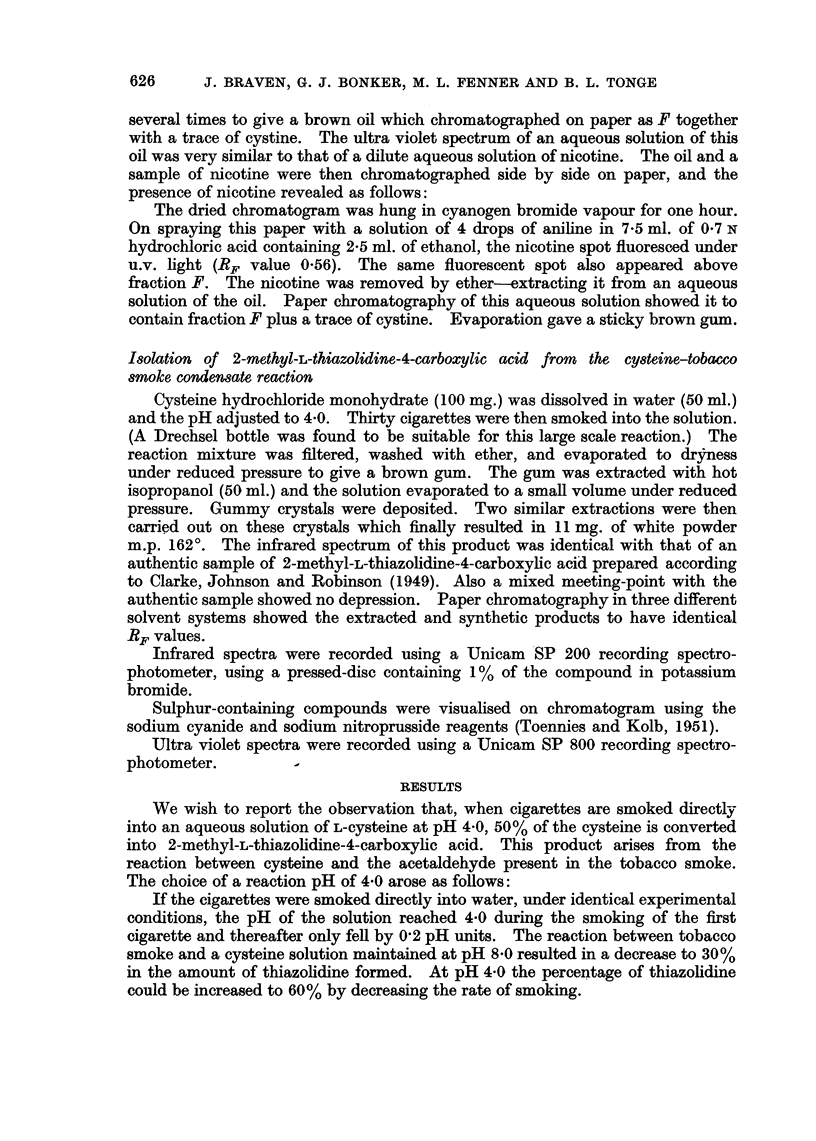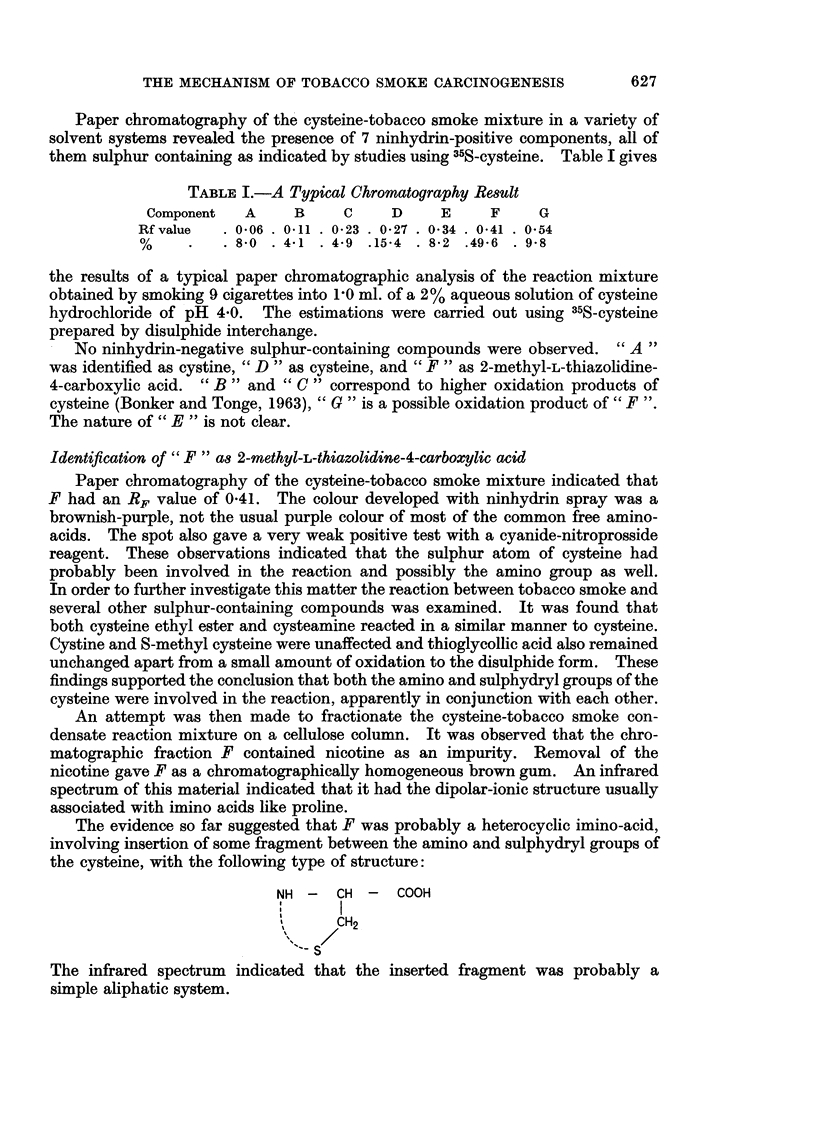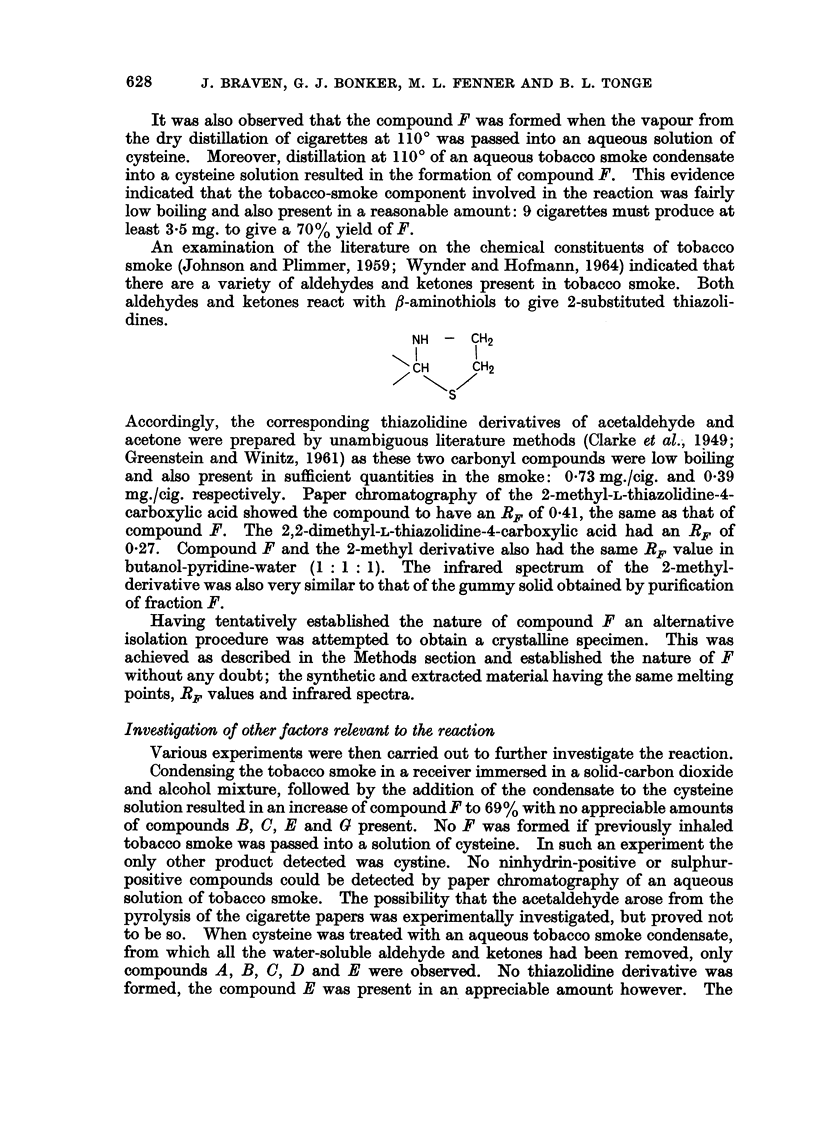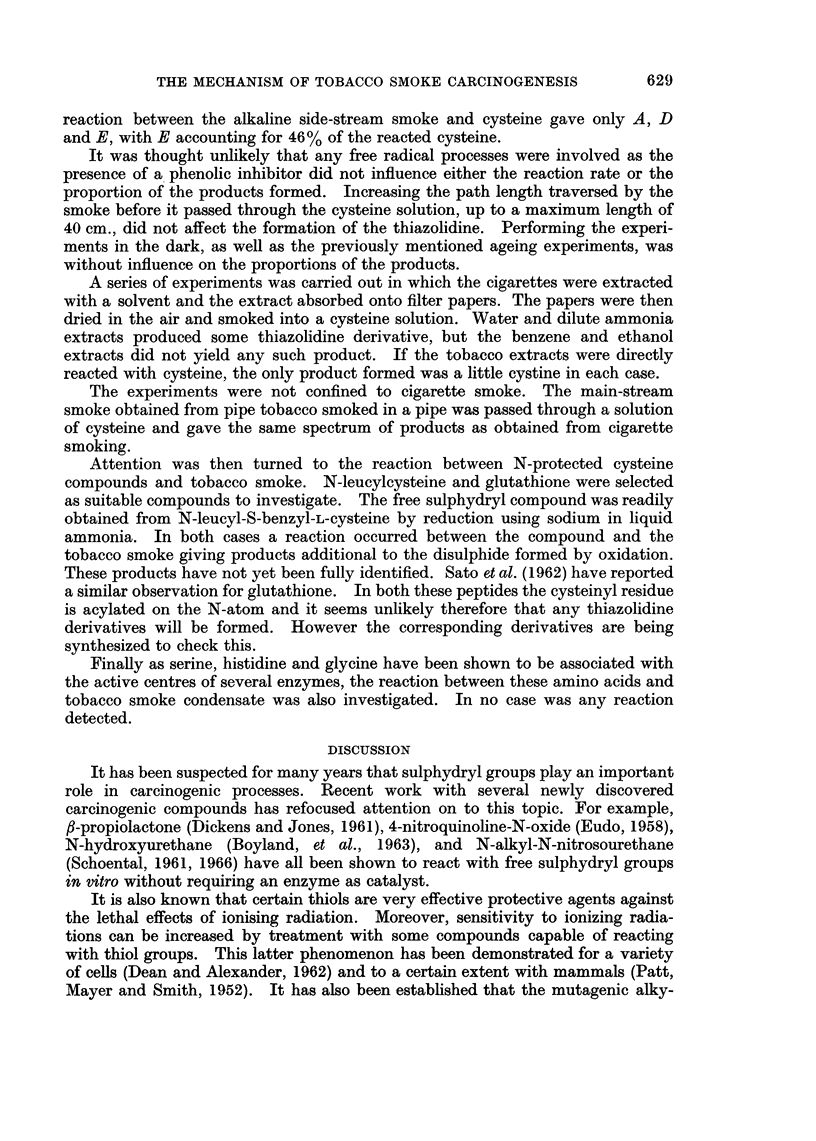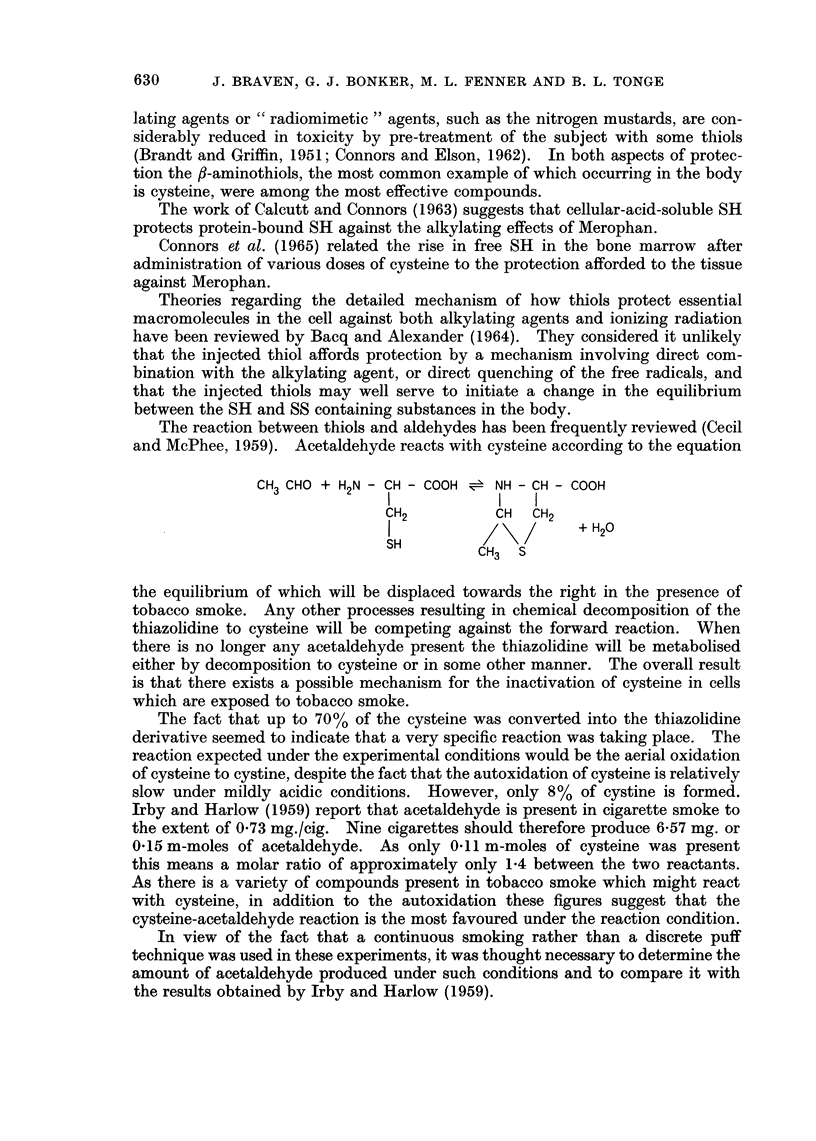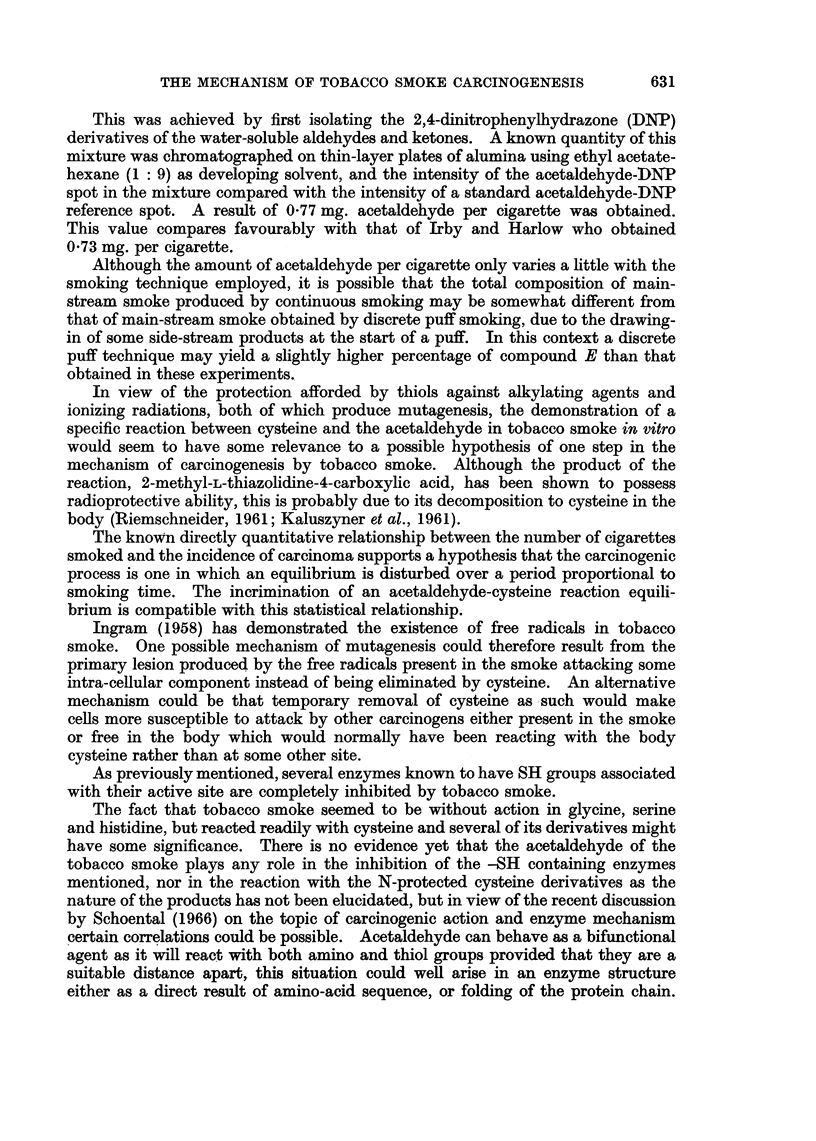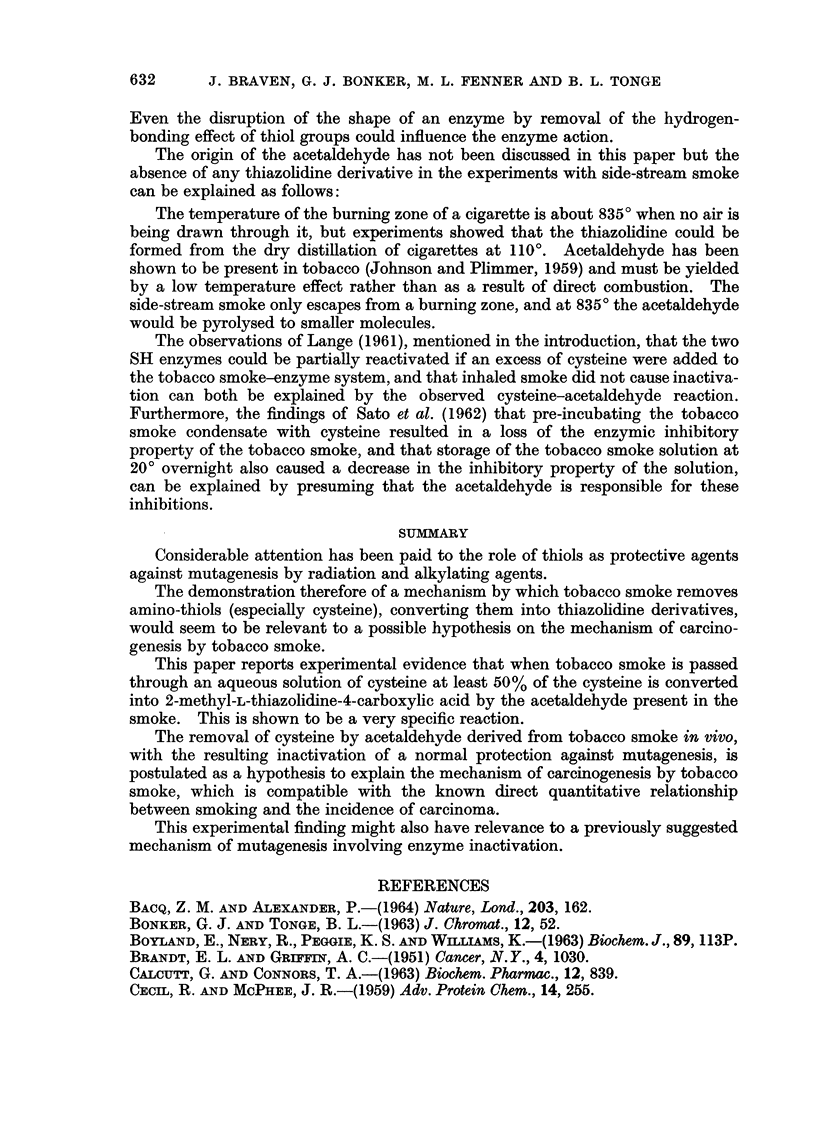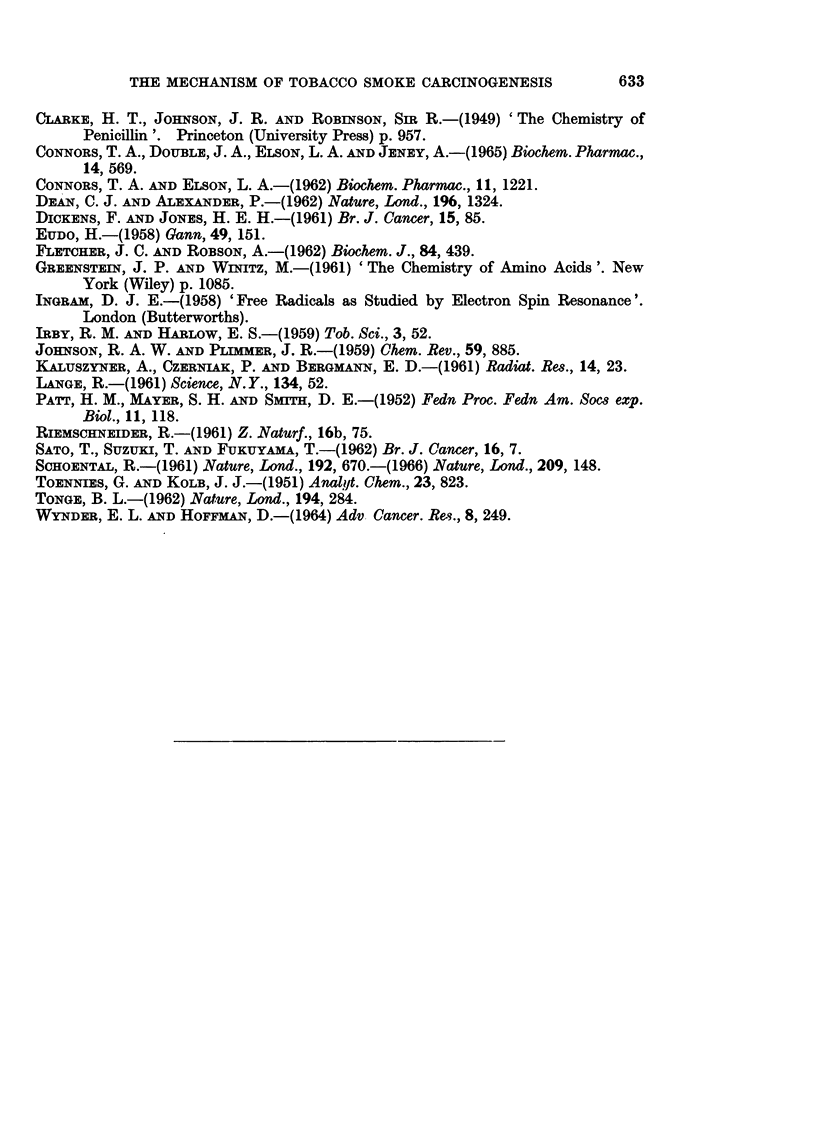# The mechanism of carcinogenesis by tobacco smoke. Some experimental observations and a hypothesis.

**DOI:** 10.1038/bjc.1967.73

**Published:** 1967-09

**Authors:** J. Braven, G. J. Bonker, M. L. Fenner, B. L. Tonge


### THE MECHANISM OF CARCINOGENESIS BY TOBACCO SMOKE

### Some Experimental Observations and a Hypothesis

J. BRAVEN, G. J. BONKER, M. L. FENNER* and B. L. TONGE†

*From the College of Technology, Plymouth, Devon*
*The Radiotherapy Department*, Freedom Fields Hospital, Plymouth, Devon*
*and the College of Technology†, Wolverhampton, Staffordshire*

Received for publication March 29, 1967

The importance of certain thiols and disulphides as protective agents against the lethal effects of ionizing radiation, and certain alkylating agents such as nitrogen mustards is well known. Two of the most effective radiation protection agents are cysteine and cysteamine.

The amino acid cysteine occurs in the body in both the free and combined forms. Free cysteine occurs in the free state in equilibrium with the disulphide cystine, and in combination in such diverse molecular species as glutathione, proteins and enzymes. The sulphydryl groups present in molecules involved in intra-cellular metabolism have several functions, notably, participation in enzymic activity, regulation of the oxidation-reduction potential and in determining the degree of folding and coiling in the tertiary structure of enzyme and protein molecules.

In a study of the chemical aspects of carcinogenesis, Tonge (1962) had reported the effect of tobacco smoke condensate on the aerial oxidation of cysteine. Evidence was produced supporting the presence of trapped free-radicals in tobacco smoke condensate.

Lange (1961) has shown that two SH containing enzymes, rabbit muscle glyceraldehyde-3-phosphate dehydrogenase and yeast alcohol dehydrogenase were irreversibly inhibited by tobacco smoke. The addition of a large excess of cysteine caused a partial enzymic reactivation and furthermore the inhibition did not occur with previously inhaled smoke. It was possible to explain these results by the presence of peroxide arising from the free radicals present in tobacco smoke condensate.

Sato, Suzuki and Fukuyama (1962) have reported that the enzymes succinic dehydrogenase and urease could be inhibited by the water-soluble fraction of tobacco smoke; moreover, the inhibition could be reduced almost to zero by pre-incubating the tobacco smoke condensate with cysteine or glutathione. The process of inhibition was markedly decreased if the tobacco smoke solutions were left at 20° C. overnight before reaction with the enzymes. They also reported that both cysteine and glutathione reacted with the water-soluble fraction of tobacco smoke, but no reaction products were identified.

In view therefore of the established relationship between excessive cigarette smoking and carcinoma of the bronchus it was decided to investigate the action of cigarette smoke on cysteine in more detail than hitherto.

### METHODS

### *Standard smoking procedure*

L-cysteine hydrochloride monohydrate (20 mg. 0·11 m-moles) was dissolved in water (1·0 ml.) and the pH of the solution adjusted to 4·0 by the cautious addition of ammonia solution from a capillary tube. Cigarettes were then smoked one at a time directly into the solution using the apparatus illustrated in Fig. 1. Air

FIG. 1.—Apparatus used for the direct smoking of cigarettes into an aqueous solution of cysteine.

was drawn through the cigarette at a steady rate by means of an aspirator thus maintaining even smoking. Each cigarette was smoked for 6 minutes, and on completion was immediately replaced by a pre-lit cigarette thus minimising the amount of air drawn through the solution; the length of the smoked butt was approximately 0·5 cm. To follow the decrease in cysteine concentration small aliquots of the reaction mixture were withdrawn at intervals using a capillary tube, and treated with dilute ammonia solution followed by nitroprusside reagent. This test was negative after 9 cigarettes. The reaction mixture was then chromatographed on Whatman No. 1 paper, using an ascending development of *n*-butanol, acetic acid, water (60, 15, 25). The reaction products were located with ninhydrin reagent (0·2% ninhydrin in acetone).

A similar procedure was used to investigate the action of tobacco smoke condensate on the other substances mentioned in this work.

In the case of S-protected substances 9 cigarettes were smoked into 0·11 m-moles of the compound dissolved in 1·0 ml. of water.

*Quantitative analysis of the cysteine-tobacco smoke reaction using $^{35}$S-L-cysteine.*

The radioactive materials were supplied by the Radiochemical Centre, Amersham, Bucks, England.

$^{35}$S-cysteine hydrochloride was found to undergo appreciable radiolysis on storage, even in acid solution at temperatures near 0°, whereas $^{35}$S-cysteine hydrochloride stored under nitrogen, was considerably more stable over long periods under the same conditions (Fletcher and Robson, 1962; Ogle, 1963, personal communication). On account of this the $^{35}$S-L-cysteine was freshly prepared before each experiment by a disulphide exchange reaction between L-cysteine hydrochloride and $^{35}$S-L-cystine as follows: $^{35}$S-cystine (3·4 mg.; 0·62 mc) was dissolved in 1·0 ml. of 0·5 N hydrochloric acid. This was used as a stock solution for a series of experiments and always chromatographed before use to check against possible degradation by radiolysis during storage at 0°. L-cysteine hydrochloride monohydrate (20 mg.; 0·11 m-moles) was dissolved in water (1·0 ml.) and 50 $\mu$l. of the stock solution of $^{35}$S-cystine added. The pH was then adjusted to 10·0 with ammonia solution and the reaction left for 2 minutes to reach equilibrium. The pH was then readjusted to 4·0 with conc. hydrochloric acid and tobacco smoke passed through in the previously described manner. The reaction mixture was then chromatographed.

Radioactive substances on the chromatograms were located by autoradiography using " Kodirex X-ray film " (Kodak Limited, England), exposed for a period of 48 hours. The film was protected from acidic materials on the paper by thin polythene sheets. The developed film was then again placed in contact with the chromatogram, the two sheets mounted on a " light-box " and the outline of the radioactive areas on the chromatography paper marked in pencil. These areas were then cut out and the activity of each spot estimated with a proportionality counter. The time for about 10,000 " counts " was noted and the quotient then expressed as " counts " per minute. The proportion of each reaction product was then expressed as a simple percentage. There were no ninhydrin-positive components which did not contain sulphur. A typical set of results is given in Table I.

The method was considered sufficiently accurate as in a control experiment $^{35}$S-cysteine spots with relative intensities of 1·0, 4·0 and 6·0 gave an experimental ratio of 1·0, 4·04 and 6·03.

*Fractionation of the cysteine-tobacco smoke condensate reaction mixture*

The reaction mixture obtained by smoking 15 cigarettes into 100 mg. cysteine dissolved in 4 ml. of water was chromatographed on a column of cellulose powder (2 cm. × 30 cm.) (Whatman coarse-grade) using butanol, acetic acid, water mixture (60 : 15 : 25) as eluting solvent. 3 ml. fractions were collected using an automatic fraction collector and the contents of the tubes examined by paper chromatography. Pure fraction *F* was found in five consecutive tubes, the united contents of which were then cautiously evaporated to dryness using a rotary evaporator. The resulting oil was redissolved in water and the solution evaporated several times to give a brown oil which chromatographed on paper as *F* together with a trace of cystine. The ultra violet spectrum of an aqueous solution of this oil was very similar to that of a dilute aqueous solution of nicotine. The oil and a sample of nicotine were then chromatographed side by side on paper, and the presence of nicotine revealed as follows:

The dried chromatogram was hung in cyanogen bromide vapour for one hour. On spraying this paper with a solution of 4 drops of aniline in 7·5 ml. of 0·7 N hydrochloric acid containing 2·5 ml. of ethanol, the nicotine spot fluoresced under u.v. light ($R_F$ value 0·56). The same fluorescent spot also appeared above fraction *F*. The nicotine was removed by ether—extracting it from an aqueous solution of the oil. Paper chromatography of this aqueous solution showed it to contain fraction *F* plus a trace of cystine. Evaporation gave a sticky brown gum.

*Isolation of 2-methyl-L-thiazolidine-4-carboxylic acid from the cysteine–tobacco smoke condensate reaction*

Cysteine hydrochloride monohydrate (100 mg.) was dissolved in water (50 ml.) and the pH adjusted to 4·0. Thirty cigarettes were then smoked into the solution. (A Drechsel bottle was found to be suitable for this large scale reaction.) The reaction mixture was filtered, washed with ether, and evaporated to dryness under reduced pressure to give a brown gum. The gum was extracted with hot isopropanol (50 ml.) and the solution evaporated to a small volume under reduced pressure. Gummy crystals were deposited. Two similar extractions were then carried out on these crystals which finally resulted in 11 mg. of white powder m.p. 162°. The infrared spectrum of this product was identical with that of an authentic sample of 2-methyl-L-thiazolidine-4-carboxylic acid prepared according to Clarke, Johnson and Robinson (1949). Also a mixed meeting-point with the authentic sample showed no depression. Paper chromatography in three different solvent systems showed the extracted and synthetic products to have identical $R_F$ values.

Infrared spectra were recorded using a Unicam SP 200 recording spectrophotometer, using a pressed-disc containing 1% of the compound in potassium bromide.

Sulphur-containing compounds were visualised on chromatogram using the sodium cyanide and sodium nitroprusside reagents (Toennies and Kolb, 1951).

Ultra violet spectra were recorded using a Unicam SP 800 recording spectrophotometer.

### RESULTS

We wish to report the observation that, when cigarettes are smoked directly into an aqueous solution of L-cysteine at pH 4·0, 50% of the cysteine is converted into 2-methyl-L-thiazolidine-4-carboxylic acid. This product arises from the reaction between cysteine and the acetaldehyde present in the tobacco smoke. The choice of a reaction pH of 4·0 arose as follows:

If the cigarettes were smoked directly into water, under identical experimental conditions, the pH of the solution reached 4·0 during the smoking of the first cigarette and thereafter only fell by 0·2 pH units. The reaction between tobacco smoke and a cysteine solution maintained at pH 8·0 resulted in a decrease to 30% in the amount of thiazolidine formed. At pH 4·0 the percentage of thiazolidine could be increased to 60% by decreasing the rate of smoking.

Paper chromatography of the cysteine-tobacco smoke mixture in a variety of solvent systems revealed the presence of 7 ninhydrin-positive components, all of them sulphur containing as indicated by studies using $^{35}$S-cysteine. Table I gives

TABLE I.—*A Typical Chromatography Result*

| Component | A | B | C | D | E | F | G |
|---|---|---|---|---|---|---|---|
| Rf value | 0·06 | 0·11 | 0·23 | 0·27 | 0·34 | 0·41 | 0·54 |
| % | 8·0 | 4·1 | 4·9 | 15·4 | 8·2 | 49·6 | 9·8 |

the results of a typical paper chromatographic analysis of the reaction mixture obtained by smoking 9 cigarettes into 1·0 ml. of a 2% aqueous solution of cysteine hydrochloride of pH 4·0. The estimations were carried out using $^{35}$S-cysteine prepared by disulphide interchange.

No ninhydrin-negative sulphur-containing compounds were observed. " *A* " was identified as cystine, " *D* " as cysteine, and " *F* " as 2-methyl-L-thiazolidine-4-carboxylic acid. " *B* " and " *C* " correspond to higher oxidation products of cysteine (Bonker and Tonge, 1963), " *G* " is a possible oxidation product of " *F* ". The nature of " *E* " is not clear.

*Identification of " F " as 2-methyl-*L*-thiazolidine-4-carboxylic acid*

Paper chromatography of the cysteine-tobacco smoke mixture indicated that *F* had an $R_F$ value of 0·41. The colour developed with ninhydrin spray was a brownish-purple, not the usual purple colour of most of the common free amino-acids. The spot also gave a very weak positive test with a cyanide-nitroprosside reagent. These observations indicated that the sulphur atom of cysteine had probably been involved in the reaction and possibly the amino group as well. In order to further investigate this matter the reaction between tobacco smoke and several other sulphur-containing compounds was examined. It was found that both cysteine ethyl ester and cysteamine reacted in a similar manner to cysteine. Cystine and S-methyl cysteine were unaffected and thioglycollic acid also remained unchanged apart from a small amount of oxidation to the disulphide form. These findings supported the conclusion that both the amino and sulphydryl groups of the cysteine were involved in the reaction, apparently in conjunction with each other.

An attempt was then made to fractionate the cysteine-tobacco smoke condensate reaction mixture on a cellulose column. It was observed that the chromatographic fraction *F* contained nicotine as an impurity. Removal of the nicotine gave *F* as a chromatographically homogeneous brown gum. An infrared spectrum of this material indicated that it had the dipolar-ionic structure usually associated with imino acids like proline.

The evidence so far suggested that *F* was probably a heterocyclic imino-acid, involving insertion of some fragment between the amino and sulphydryl groups of the cysteine, with the following type of structure:

The infrared spectrum indicated that the inserted fragment was probably a simple aliphatic system.

It was also observed that the compound *F* was formed when the vapour from the dry distillation of cigarettes at 110° was passed into an aqueous solution of cysteine. Moreover, distillation at 110° of an aqueous tobacco smoke condensate into a cysteine solution resulted in the formation of compound *F*. This evidence indicated that the tobacco-smoke component involved in the reaction was fairly low boiling and also present in a reasonable amount: 9 cigarettes must produce at least 3·5 mg. to give a 70% yield of *F*.

An examination of the literature on the chemical constituents of tobacco smoke (Johnson and Plimmer, 1959; Wynder and Hofmann, 1964) indicated that there are a variety of aldehydes and ketones present in tobacco smoke. Both aldehydes and ketones react with β-aminothiols to give 2-substituted thiazolidines.

Accordingly, the corresponding thiazolidine derivatives of acetaldehyde and acetone were prepared by unambiguous literature methods (Clarke *et al.*, 1949; Greenstein and Winitz, 1961) as these two carbonyl compounds were low boiling and also present in sufficient quantities in the smoke: 0·73 mg./cig. and 0·39 mg./cig. respectively. Paper chromatography of the 2-methyl-L-thiazolidine-4-carboxylic acid showed the compound to have an $R_F$ of 0·41, the same as that of compound *F*. The 2,2-dimethyl-L-thiazolidine-4-carboxylic acid had an $R_F$ of 0·27. Compound *F* and the 2-methyl derivative also had the same $R_F$ value in butanol-pyridine-water (1 : 1 : 1). The infrared spectrum of the 2-methyl-derivative was also very similar to that of the gummy solid obtained by purification of fraction *F*.

Having tentatively established the nature of compound *F* an alternative isolation procedure was attempted to obtain a crystalline specimen. This was achieved as described in the Methods section and established the nature of *F* without any doubt; the synthetic and extracted material having the same melting points, $R_F$ values and infrared spectra.

*Investigation of other factors relevant to the reaction*

Various experiments were then carried out to further investigate the reaction.

Condensing the tobacco smoke in a receiver immersed in a solid-carbon dioxide and alcohol mixture, followed by the addition of the condensate to the cysteine solution resulted in an increase of compound *F* to 69% with no appreciable amounts of compounds *B*, *C*, *E* and *G* present. No *F* was formed if previously inhaled tobacco smoke was passed into a solution of cysteine. In such an experiment the only other product detected was cystine. No ninhydrin-positive or sulphur-positive compounds could be detected by paper chromatography of an aqueous solution of tobacco smoke. The possibility that the acetaldehyde arose from the pyrolysis of the cigarette papers was experimentally investigated, but proved not to be so. When cysteine was treated with an aqueous tobacco smoke condensate, from which all the water-soluble aldehyde and ketones had been removed, only compounds *A*, *B*, *C*, *D* and *E* were observed. No thiazolidine derivative was formed, the compound *E* was present in an appreciable amount however. The reaction between the alkaline side-stream smoke and cysteine gave only *A*, *D* and *E*, with *E* accounting for 46% of the reacted cysteine.

It was thought unlikely that any free radical processes were involved as the presence of a phenolic inhibitor did not influence either the reaction rate or the proportion of the products formed. Increasing the path length traversed by the smoke before it passed through the cysteine solution, up to a maximum length of 40 cm., did not affect the formation of the thiazolidine. Performing the experiments in the dark, as well as the previously mentioned ageing experiments, was without influence on the proportions of the products.

A series of experiments was carried out in which the cigarettes were extracted with a solvent and the extract absorbed onto filter papers. The papers were then dried in the air and smoked into a cysteine solution. Water and dilute ammonia extracts produced some thiazolidine derivative, but the benzene and ethanol extracts did not yield any such product. If the tobacco extracts were directly reacted with cysteine, the only product formed was a little cystine in each case.

The experiments were not confined to cigarette smoke. The main-stream smoke obtained from pipe tobacco smoked in a pipe was passed through a solution of cysteine and gave the same spectrum of products as obtained from cigarette smoking.

Attention was then turned to the reaction between N-protected cysteine compounds and tobacco smoke. N-leucylcysteine and glutathione were selected as suitable compounds to investigate. The free sulphydryl compound was readily obtained from N-leucyl-S-benzyl-L-cysteine by reduction using sodium in liquid ammonia. In both cases a reaction occurred between the compound and the tobacco smoke giving products additional to the disulphide formed by oxidation. These products have not yet been fully identified. Sato *et al.* (1962) have reported a similar observation for glutathione. In both these peptides the cysteinyl residue is acylated on the N-atom and it seems unlikely therefore that any thiazolidine derivatives will be formed. However the corresponding derivatives are being synthesized to check this.

Finally as serine, histidine and glycine have been shown to be associated with the active centres of several enzymes, the reaction between these amino acids and tobacco smoke condensate was also investigated. In no case was any reaction detected.

### DISCUSSION

It has been suspected for many years that sulphydryl groups play an important role in carcinogenic processes. Recent work with several newly discovered carcinogenic compounds has refocused attention on to this topic. For example, $\beta$-propiolactone (Dickens and Jones, 1961), 4-nitroquinoline-N-oxide (Eudo, 1958), N-hydroxyurethane (Boyland, *et al.*, 1963), and N-alkyl-N-nitrosourethane (Schoental, 1961, 1966) have all been shown to react with free sulphydryl groups *in vitro* without requiring an enzyme as catalyst.

It is also known that certain thiols are very effective protective agents against the lethal effects of ionising radiation. Moreover, sensitivity to ionizing radiations can be increased by treatment with some compounds capable of reacting with thiol groups. This latter phenomenon has been demonstrated for a variety of cells (Dean and Alexander, 1962) and to a certain extent with mammals (Patt, Mayer and Smith, 1952). It has also been established that the mutagenic alkylating agents or " radiomimetic " agents, such as the nitrogen mustards, are considerably reduced in toxicity by pre-treatment of the subject with some thiols (Brandt and Griffin, 1951; Connors and Elson, 1962). In both aspects of protection the $\beta$-aminothiols, the most common example of which occurring in the body is cysteine, were among the most effective compounds.

The work of Calcutt and Connors (1963) suggests that cellular-acid-soluble SH protects protein-bound SH against the alkylating effects of Merophan.

Connors *et al.* (1965) related the rise in free SH in the bone marrow after administration of various doses of cysteine to the protection afforded to the tissue against Merophan.

Theories regarding the detailed mechanism of how thiols protect essential macromolecules in the cell against both alkylating agents and ionizing radiation have been reviewed by Bacq and Alexander (1964). They considered it unlikely that the injected thiol affords protection by a mechanism involving direct combination with the alkylating agent, or direct quenching of the free radicals, and that the injected thiols may well serve to initiate a change in the equilibrium between the SH and SS containing substances in the body.

The reaction between thiols and aldehydes has been frequently reviewed (Cecil and McPhee, 1959). Acetaldehyde reacts with cysteine according to the equation the equilibrium of which will be displaced towards the right in the presence of tobacco smoke. Any other processes resulting in chemical decomposition of the thiazolidine to cysteine will be competing against the forward reaction. When there is no longer any acetaldehyde present the thiazolidine will be metabolised either by decomposition to cysteine or in some other manner. The overall result is that there exists a possible mechanism for the inactivation of cysteine in cells which are exposed to tobacco smoke.

The fact that up to 70% of the cysteine was converted into the thiazolidine derivative seemed to indicate that a very specific reaction was taking place. The reaction expected under the experimental conditions would be the aerial oxidation of cysteine to cystine, despite the fact that the autoxidation of cysteine is relatively slow under mildly acidic conditions. However, only 8% of cystine is formed. Irby and Harlow (1959) report that acetaldehyde is present in cigarette smoke to the extent of 0·73 mg./cig. Nine cigarettes should therefore produce 6·57 mg. or 0·15 m-moles of acetaldehyde. As only 0·11 m-moles of cysteine was present this means a molar ratio of approximately only 1·4 between the two reactants. As there is a variety of compounds present in tobacco smoke which might react with cysteine, in addition to the autoxidation these figures suggest that the cysteine-acetaldehyde reaction is the most favoured under the reaction condition.

In view of the fact that a continuous smoking rather than a discrete puff technique was used in these experiments, it was thought necessary to determine the amount of acetaldehyde produced under such conditions and to compare it with the results obtained by Irby and Harlow (1959).

This was achieved by first isolating the 2,4-dinitrophenylhydrazone (DNP) derivatives of the water-soluble aldehydes and ketones. A known quantity of this mixture was chromatographed on thin-layer plates of alumina using ethyl acetate-hexane (1 : 9) as developing solvent, and the intensity of the acetaldehyde-DNP spot in the mixture compared with the intensity of a standard acetaldehyde-DNP reference spot. A result of 0·77 mg. acetaldehyde per cigarette was obtained. This value compares favourably with that of Irby and Harlow who obtained 0·73 mg. per cigarette.

Although the amount of acetaldehyde per cigarette only varies a little with the smoking technique employed, it is possible that the total composition of main-stream smoke produced by continuous smoking may be somewhat different from that of main-stream smoke obtained by discrete puff smoking, due to the drawing-in of some side-stream products at the start of a puff. In this context a discrete puff technique may yield a slightly higher percentage of compound *E* than that obtained in these experiments.

In view of the protection afforded by thiols against alkylating agents and ionizing radiations, both of which produce mutagenesis, the demonstration of a specific reaction between cysteine and the acetaldehyde in tobacco smoke *in vitro* would seem to have some relevance to a possible hypothesis of one step in the mechanism of carcinogenesis by tobacco smoke. Although the product of the reaction, 2-methyl-L-thiazolidine-4-carboxylic acid, has been shown to possess radioprotective ability, this is probably due to its decomposition to cysteine in the body (Riemschneider, 1961; Kaluszyner *et al.*, 1961).

The known directly quantitative relationship between the number of cigarettes smoked and the incidence of carcinoma supports a hypothesis that the carcinogenic process is one in which an equilibrium is disturbed over a period proportional to smoking time. The incrimination of an acetaldehyde-cysteine reaction equilibrium is compatible with this statistical relationship.

Ingram (1958) has demonstrated the existence of free radicals in tobacco smoke. One possible mechanism of mutagenesis could therefore result from the primary lesion produced by the free radicals present in the smoke attacking some intra-cellular component instead of being eliminated by cysteine. An alternative mechanism could be that temporary removal of cysteine as such would make cells more susceptible to attack by other carcinogens either present in the smoke or free in the body which would normally have been reacting with the body cysteine rather than at some other site.

As previously mentioned, several enzymes known to have SH groups associated with their active site are completely inhibited by tobacco smoke.

The fact that tobacco smoke seemed to be without action in glycine, serine and histidine, but reacted readily with cysteine and several of its derivatives might have some significance. There is no evidence yet that the acetaldehyde of the tobacco smoke plays any role in the inhibition of the –SH containing enzymes mentioned, nor in the reaction with the N-protected cysteine derivatives as the nature of the products has not been elucidated, but in view of the recent discussion by Schoental (1966) on the topic of carcinogenic action and enzyme mechanism certain correlations could be possible. Acetaldehyde can behave as a bifunctional agent as it will react with both amino and thiol groups provided that they are a suitable distance apart, this situation could well arise in an enzyme structure either as a direct result of amino-acid sequence, or folding of the protein chain.

Even the disruption of the shape of an enzyme by removal of the hydrogen-bonding effect of thiol groups could influence the enzyme action.

The origin of the acetaldehyde has not been discussed in this paper but the absence of any thiazolidine derivative in the experiments with side-stream smoke can be explained as follows:

The temperature of the burning zone of a cigarette is about 835° when no air is being drawn through it, but experiments showed that the thiazolidine could be formed from the dry distillation of cigarettes at 110°. Acetaldehyde has been shown to be present in tobacco (Johnson and Plimmer, 1959) and must be yielded by a low temperature effect rather than as a result of direct combustion. The side-stream smoke only escapes from a burning zone, and at 835° the acetaldehyde would be pyrolysed to smaller molecules.

The observations of Lange (1961), mentioned in the introduction, that the two SH enzymes could be partially reactivated if an excess of cysteine were added to the tobacco smoke–enzyme system, and that inhaled smoke did not cause inactivation can both be explained by the observed cysteine–acetaldehyde reaction. Furthermore, the findings of Sato *et al.* (1962) that pre-incubating the tobacco smoke condensate with cysteine resulted in a loss of the enzymic inhibitory property of the tobacco smoke, and that storage of the tobacco smoke solution at 20° overnight also caused a decrease in the inhibitory property of the solution, can be explained by presuming that the acetaldehyde is responsible for these inhibitions.

### SUMMARY

Considerable attention has been paid to the role of thiols as protective agents against mutagenesis by radiation and alkylating agents.

The demonstration therefore of a mechanism by which tobacco smoke removes amino-thiols (especially cysteine), converting them into thiazolidine derivatives, would seem to be relevant to a possible hypothesis on the mechanism of carcinogenesis by tobacco smoke.

This paper reports experimental evidence that when tobacco smoke is passed through an aqueous solution of cysteine at least 50% of the cysteine is converted into 2-methyl-L-thiazolidine-4-carboxylic acid by the acetaldehyde present in the smoke. This is shown to be a very specific reaction.

The removal of cysteine by acetaldehyde derived from tobacco smoke *in vivo*, with the resulting inactivation of a normal protection against mutagenesis, is postulated as a hypothesis to explain the mechanism of carcinogenesis by tobacco smoke, which is compatible with the known direct quantitative relationship between smoking and the incidence of carcinoma.

This experimental finding might also have relevance to a previously suggested mechanism of mutagenesis involving enzyme inactivation.

### REFERENCES

Bacq, Z. M. and Alexander, P.—(1964) *Nature, Lond.*, **203**, 162.
Bonker, G. J. and Tonge, B. L.—(1963) *J. Chromat.*, **12**, 52.
Boyland, E., Nery, R., Peggie, K. S. and Williams, K.—(1963) *Biochem. J.*, **89**, 113P.
Brandt, E. L. and Griffin, A. C.—(1951) *Cancer, N.Y.*, **4**, 1030.
Calcutt, G. and Connors, T. A.—(1963) *Biochem. Pharmac.*, **12**, 839.
Cecil, R. and McPhee, J. R.—(1959) *Adv. Protein Chem.*, **14**, 255.

CLARKE, H. T., JOHNSON, J. R. AND ROBINSON, SIR R.—(1949) 'The Chemistry of Penicillin'. Princeton (University Press) p. 957.
CONNORS, T. A., DOUBLE, J. A., ELSON, L. A. AND JENEY, A.—(1965) *Biochem. Pharmac.*, **14**, 569.
CONNORS, T. A. AND ELSON, L. A.—(1962) *Biochem. Pharmac.*, **11**, 1221.
DEAN, C. J. AND ALEXANDER, P.—(1962) *Nature, Lond.*, **196**, 1324.
DICKENS, F. AND JONES, H. E. H.—(1961) *Br. J. Cancer*, **15**, 85.
EUDO, H.—(1958) *Gann*, **49**, 151.
FLETCHER, J. C. AND ROBSON, A.—(1962) *Biochem. J.*, **84**, 439.
GREENSTEIN, J. P. AND WINITZ, M.—(1961) 'The Chemistry of Amino Acids'. New York (Wiley) p. 1085.
INGRAM, D. J. E.—(1958) 'Free Radicals as Studied by Electron Spin Resonance'. London (Butterworths).
IRBY, R. M. AND HARLOW, E. S.—(1959) *Tob. Sci.*, **3**, 52.
JOHNSON, R. A. W. AND PLIMMER, J. R.—(1959) *Chem. Rev.*, **59**, 885.
KALUSZYNER, A., CZERNIAK, P. AND BERGMANN, E. D.—(1961) *Radiat. Res.*, **14**, 23.
LANGE, R.—(1961) *Science, N.Y.*, **134**, 52.
PATT, H. M., MAYER, S. H. AND SMITH, D. E.—(1952) *Fedn Proc. Fedn Am. Socs exp. Biol.*, **11**, 118.
RIEMSCHNEIDER, R.—(1961) *Z. Naturf.*, **16**b, 75.
SATO, T., SUZUKI, T. AND FUKUYAMA, T.—(1962) *Br. J. Cancer*, **16**, 7.
SCHOENTAL, R.—(1961) *Nature, Lond.*, **192**, 670.—(1966) *Nature, Lond.*, **209**, 148.
TOENNIES, G. AND KOLB, J. J.—(1951) *Analyt. Chem.*, **23**, 823.
TONGE, B. L.—(1962) *Nature, Lond.*, **194**, 284.
WYNDER, E. L. AND HOFFMAN, D.—(1964) *Adv Cancer. Res.*, **8**, 249.